# Bioimpedance measurement: a non-invasive diagnosis of limb compartment syndrome

**DOI:** 10.3389/fbioe.2024.1433284

**Published:** 2024-08-23

**Authors:** Martin Novak, Vladimir Jecminek, Leopold Pleva, Marek Penhaker, Martin Schmidt, Tomas Mimra, Jan Kubicek, Martin Augustynek

**Affiliations:** ^1^ Department of Trauma Surgery, Ostrava University Hospital and Department of Disaster Medicine, Faculty of Medicine, Ostrava, Czechia; ^2^ Moravian-Silesian Region Emergency Medical Service, Ostrava, Czechia; ^3^ Department of Cybernetics and Biomedical Engineering of the Technical University of Ostrava, Ostrava, Czechia

**Keywords:** compartment syndrome, bioimpedance, compartment syndrome bioimpedance analyzer, bioimpedance changes, saline injection

## Abstract

**Introduction:**

The methods for diagnosing compartment syndrome non-invasively remain under debate. Bioimpedance measurements offer a promising avenue in clinical practice, detecting subtle changes in organ impedance due to volume shifts. This study explores bioimpedance measurement as a novel, painless method for diagnosing compartment syndrome, potentially enabling continuous monitoring.

**Objective:**

This work aims to develop a prototype device for non-invasive diagnosis of compartment syndrome based on bioimpedance changes and assess initial results through *in vitro* experiments on inanimate biological material. We assume a change in the bioimpedance value after the application of physiological solution.

**Materials and Methods:**

Between 2018 and 2022, a prototype device for diagnosing limb compartment syndrome was collaboratively developed with the Department of Cybernetics and Biomedical Engineering at the Technical University of Ostrava, Czech Republic. This device operates by comparing bioimpedance between two compartments, one of which is pathologically affected (experiencing compartment syndrome). The Bioimpedance Analyzer for Compartment Syndrome (BACS) has been utilized to conduct measurements on inanimate biological material in laboratory settings. Two samples of duck and chicken tissue, as well as piglets, were employed for these experiments. According to the size of sample was compartment syndrome simulated by injecting 20–120 mL saline into one limb (breast) while leaving the other as a control. Invasive intramuscular pressure measurements were conducted post-saline injection using a conventional device (Stryker). Changes in bioimpedance were evaluated following saline application.

**Results:**

The non-invasive bioimpedance measurement instrument has been developed. It meets the safety requirements of European standard EN 60601-1. Measurement of accuracy showed minimal deviation for both channels (1.08% for the left channel and 1.84% for the right channel) when measuring on resistors. Ten measurements were conducted using the BACS prototype - two on chicken legs, two on duck breasts, two on duck legs, and four on piglets. Compartment syndrome simulation was achieved for all 10 measurements (IMP variance 31–45 mmHg). Following saline application, a notable decrease in bioimpedance was observed in the compartment simulating compartment syndrome (decrease by 12–78 Ω).

**Conclusion:**

Non-invasive methods could revolutionize limb compartment syndrome diagnosis, offering advantages such as non-invasiveness and continuous monitoring of compartment swelling.

## Introduction

Compartment syndrome arises when increased pressure within a confined space restricts blood supply to nerves and muscles, exacerbating swelling (Mubarak et al., 1978). Diagnosis relies on clinical assessment, including patient-reported symptoms and objective signs. In case of uncertainty, intramuscular pressure measurement is considered the gold standard, despite its invasive nature and associated risks such as pain, bleeding, risk of infection (Jagminas, 2022).

Non-invasive diagnosis of compartment syndrome remains a topic of research and debate, lacking a universally accepted “gold standard” method (Sellei et al., 2021). Current investigations explore various techniques, including infrared imaging, laser flowmetry, near-infrared spectroscopy (NIRS), tissue stiffness measurements, pulsed wave ultrasonography, and ultrasound elastography (Sellei et al., 2021).

Bioimpedance, or the body’s resistance to electric current, is utilized in clinical settings to detect minute changes in organ impedance over time, such as in the heart, lungs, and blood vessels (Piuzzi et al., 2018). Trauma-induced structural alterations in limb soft tissues, such as swelling and bleeding, lead to volume changes, affecting bioimpedance (Kassanos, 2021).

## Objective

Development of a prototype device for non-invasive diagnosis of compartment syndrome. Evaluation of the possibilities of measuring bioimpedance in a non-invasive way in an *in vitro* experiment on inanimate biological material.

## Material and methods

### Development of the device prototype

The research was conducted in collaboration with the Department of Cybernetics and Biomedical Engineering at the Technical University of Ostrava between 2018 and 2022. The data presented in this publication were obtained through measurements of compartment syndrome using inanimate biological materials from Bartošovice farm (Agricultural Farm Nový Jičín, Veterinary University Brno). Specifically, samples from ducks and piglets were used. These inanimate biological materials were purchased from certified suppliers specializing in research purposes. All samples were handled in accordance with established ethical guidelines and protocols to ensure the integrity and accuracy of the data collected.

The non-invasive bioimpedance measurement instrument developed was named BACS.

**FIGURE 1:**
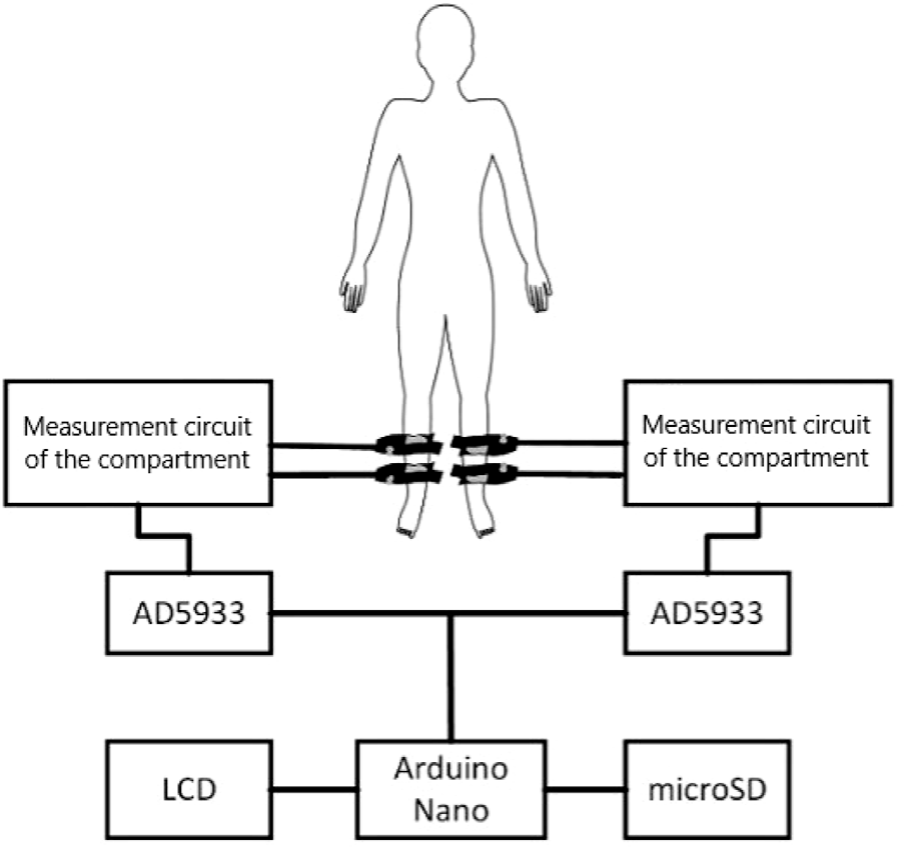
Wiring diagram of the designed BACS prototype (*source: author’s own photodocumentation*).

At its core, the instrument features two AD5933 chips serving as integrated impedance converters. Chips were supplied by Analog Devices (Wilmington, Massachusetts, United States). It can sense impedance and phase angle within a frequency range of 1–100 kHz. The dual-chip design enables simultaneous impedance sensing of the paired limbs (see Figure 1). Data acquired can be displayed on a connected computer or stored on a microSD card for later analysis.

Safety was a paramount concern during development. The instrument limits the current passing through the patient’s soft tissues using resistors, meeting the requirements of European standard EN 60601-1 issued by European Committee for Electrotechnical Standardization. To ensure measurement accuracy, the device was tested on resistors ranging from 50 to 50,000 Ω. The instrument showed a relative deviation of 1.08% for the left channel and 1.84% for the right channel when measuring on resistors. The device’s prototype was officially registered as a utility model on 16 November 2021 (utility model registration number 35 545, issued by the Industrial Property Office of the Czech Republic).

### Procedure for measuring bioimpedance with a prototype instrument

Compartment syndrome simulation involved injecting saline into one limb of the paired limbs (or one breast). Confirmation of compartment syndrome and attainment of adequate compartment pressure post-saline application were confirmed through a single measurement of intra-compartmental pressure using the Intra-compartmental Pressure Monitor system (Stryker–supplied by ProMedica Praha Group, Plc, Czech Republic).

Duck legs, breasts, chicken legs, and piglets were selected as the inanimate biological materials. The apparatus underwent testing in a laboratory environment at a consistent temperature of 25°C. Before commencing the experiment, the inanimate biological material was acclimated to room temperature for 60 min following removal from refrigeration.

Bioimpedance measurements were conducted using ECG electrodes Kendall, supplied by GPS Praha, Ltd., Czech Republic. In the duck breast experiment, Kendall H92SG ECG electrodes were initially utilized but were found to occupy excessive space and detach during use (although measurements were ultimately completed post-electrode fixation). During the bioimpedance measurements on the duck breast, also interference occurred between the individual Kendall H92SG ECG electrodes, which, due to their anatomical placement, were close together. Consequently, smaller Kendall CA610 ECG electrodes were employed in subsequent experiments from the second duck sample (two measurements) including piglets (four measurements). They occupy a smaller surface area and have greater adhesion to the inanimate biological material.

**FIGURE 2:**
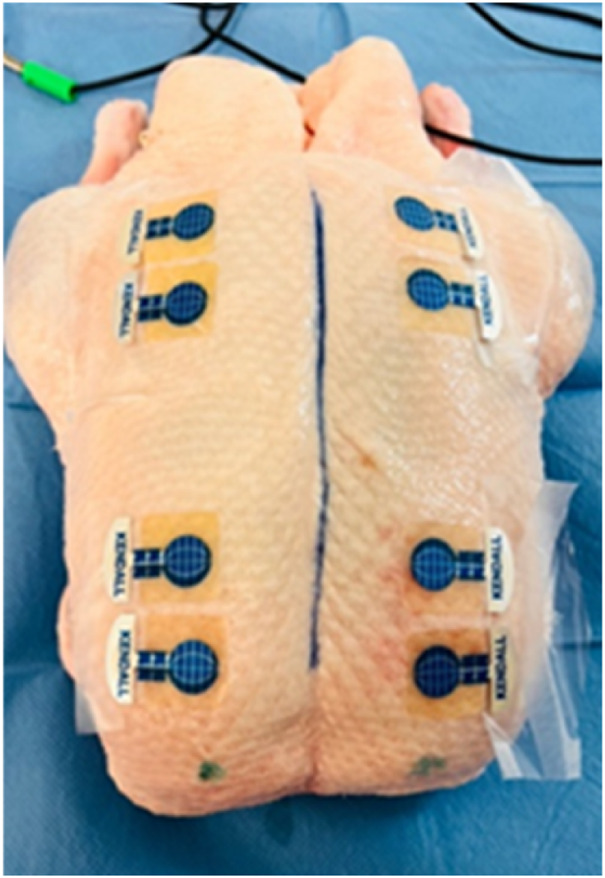
Application of Kendall CA610 electrodes to inanimate biological material (duck breast) (*source: author’s own photodocumentation*).

After preparing the inanimate biological material, electrodes were affixed to the cleaned area (see Figure 2). The placement of electrodes was mirrored to accommodate paired measurements, with four electrodes on each side.

**FIGURE 3:**
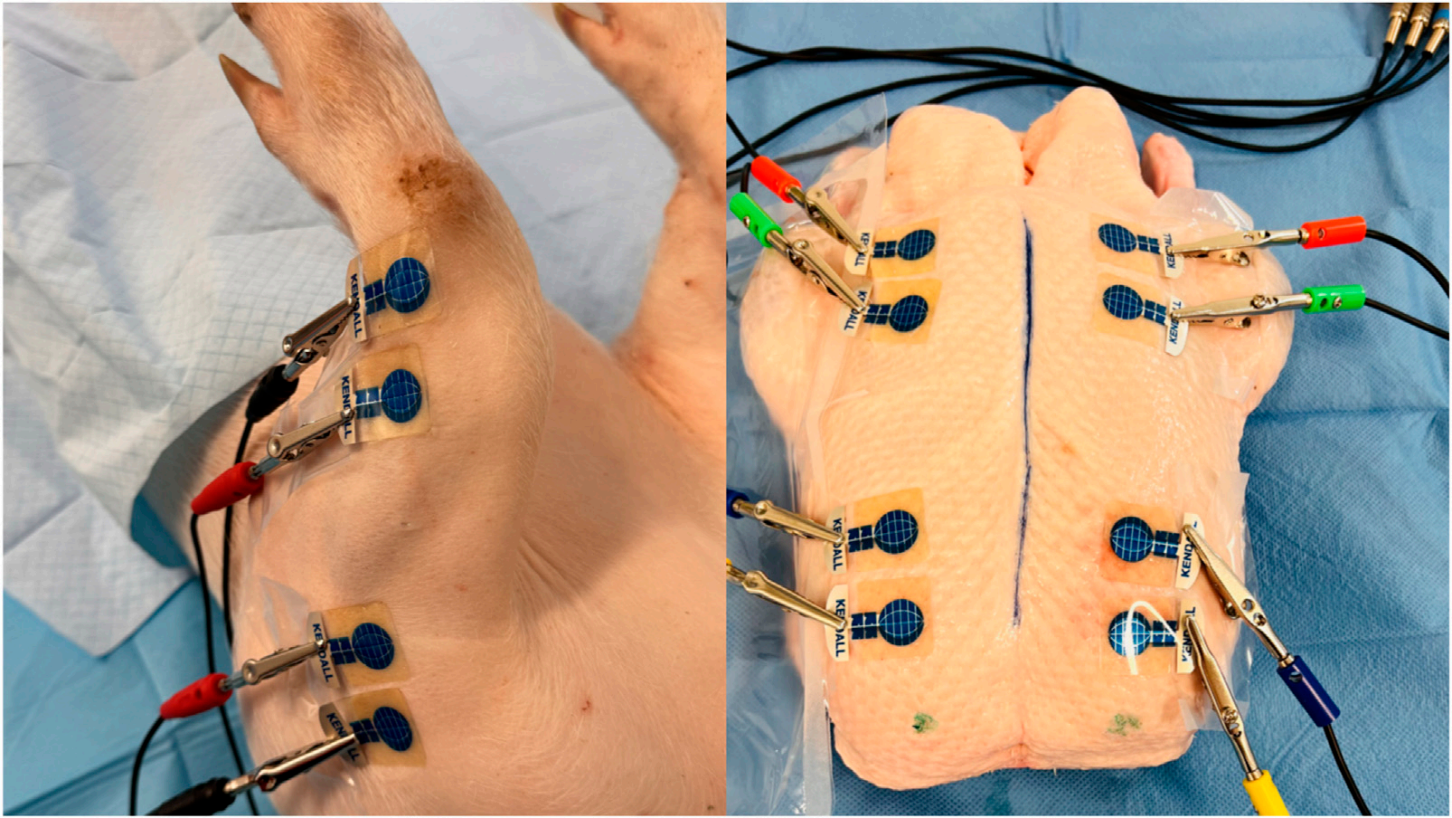
BACS measurements without changing the measured environment in a laboratory at a consistent temperature of 25°C (forelimbs of piglet and duck breasts) (*source: author’s own photodocumentation*).

**FIGURE 4:**
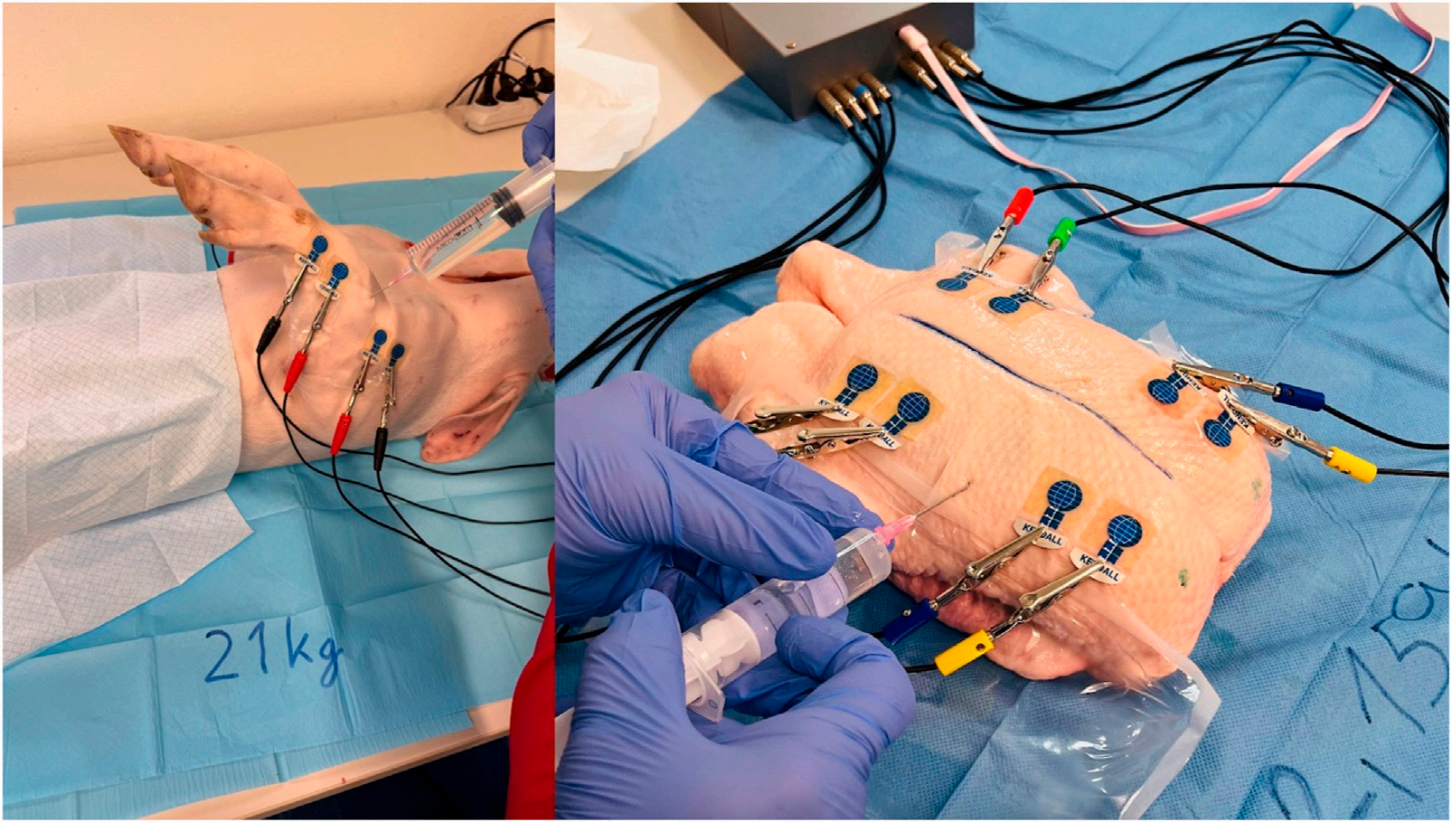
Application of saline solution to the compartment area (left forelimb arm in the piglet and left duck breast) (*source: author’s own photodocumentation)*.

**FIGURE 5:**
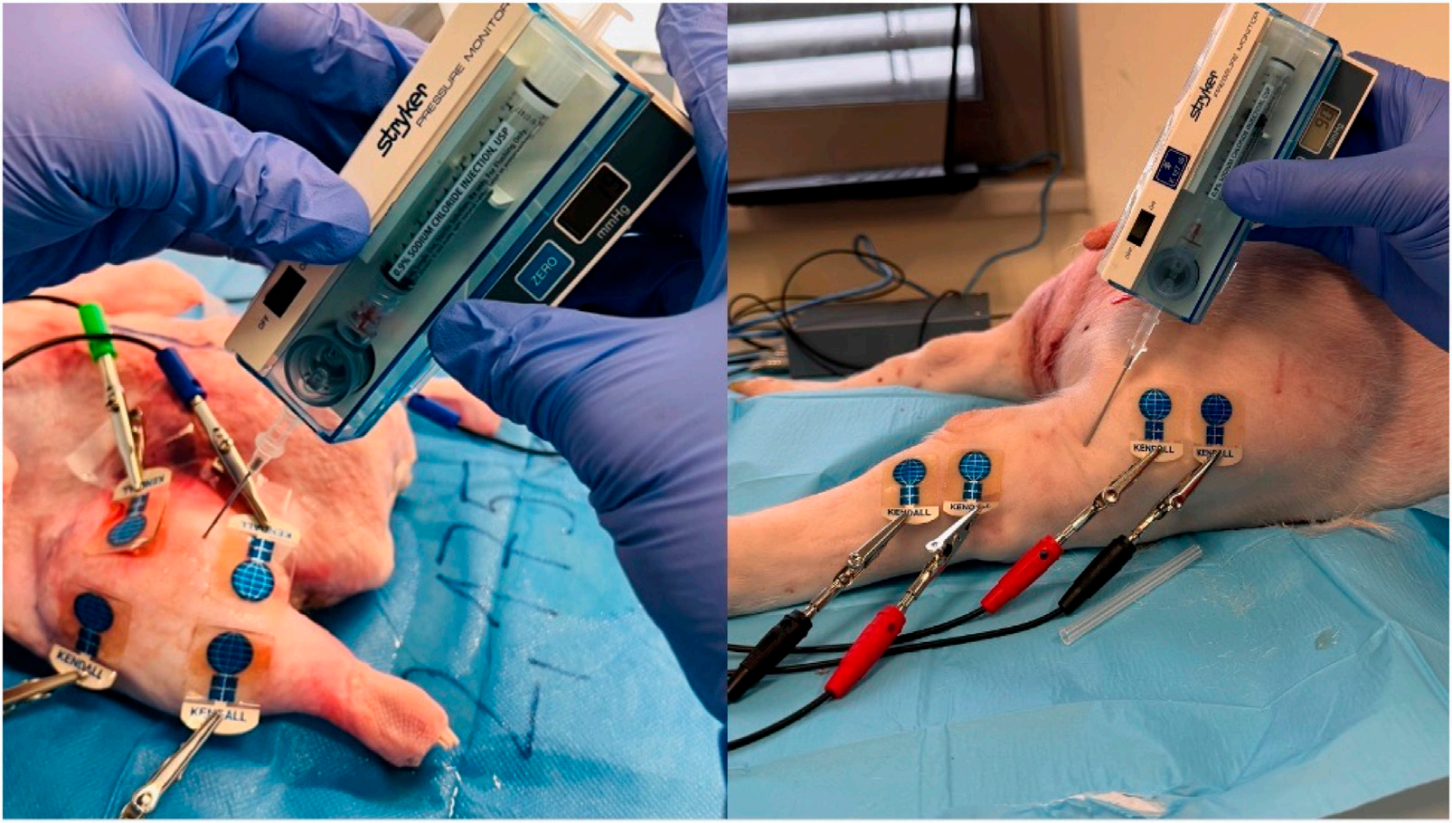
Invasive IMP measurement after saline application (left duck thigh and right hindlimb thigh in the piglet) (*source: author’s own photodocumentation*).

Following the connection of individual BACS device channels to the electrodes, basic bioimpedance measurements commenced (see Figure 3). Basic bioimpedance measurements lasted for 300 s. After this duration, physiological saline solution was introduced into the compartment space (20–40 mL for chicken legs, 80–100 mL for duck breasts, 100–120 mL for duck legs, 20–25 mL for piglet forelimb, and 50–60 mL for piglet hindlimb) (see Figure 4). Following saline application, a single invasive measurement of intramuscular pressure within the compartment was conducted (see Figure 5). The volume of saline solution administered depended on the value of intramuscular pressure (IMP) measured via invasive examination, ensuring that IMP was maintained at or above 30 mmHg.

The total measurement duration with the BACS device on both sides (with and without compartment syndrome) was 600 s. We tracked changes in bioimpedance over time by comparing compartments following physiological saline solution application to those without.

## Results

The BACS prototype was used to perform 10 measurements (2x chicken thighs, 4x duck breasts, 2x forelimb arms, 2x hindlimb thighs). In all the tested inanimate biological materials, it was possible to simulate the developing compartment syndrome by applying saline and absolute value of IMP was >30 mmHg for both samples from each inanimate biological material–chicken thighs 32, 45; duck breasts 31, 34; duck legs 36, 34; piglet forelimbs 42, 45; piglet hindlimbs 35, 38 mmHg (IMP variance 31–45 mmHg).

**FIGURE 6:**
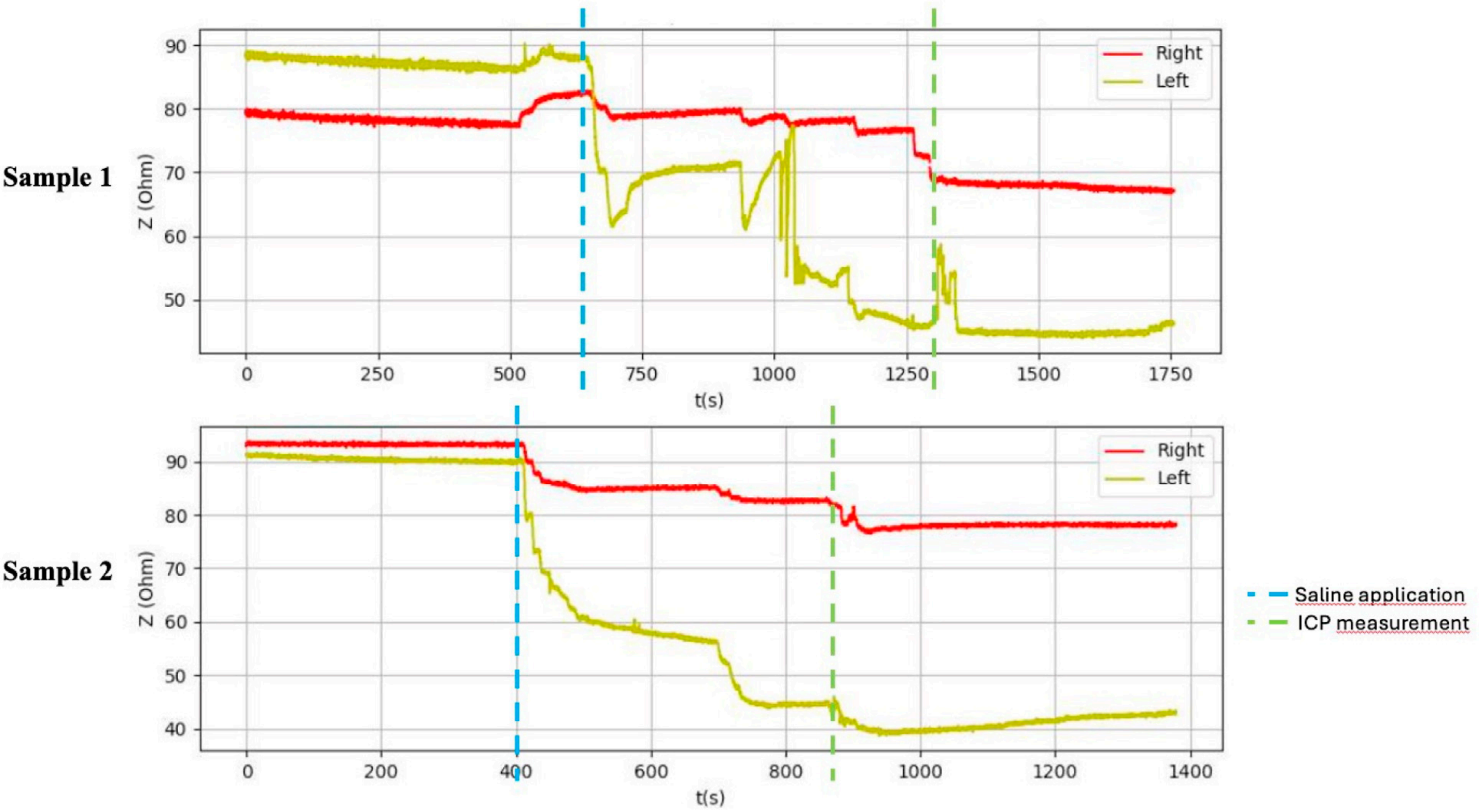
Graphical representation of the BACS measurement process (duck breasts), interference during the measurement with Kendall H92SG electrodes for sample 1, without interference using CA610 electrodes for sample 2.

**FIGURE 7:**
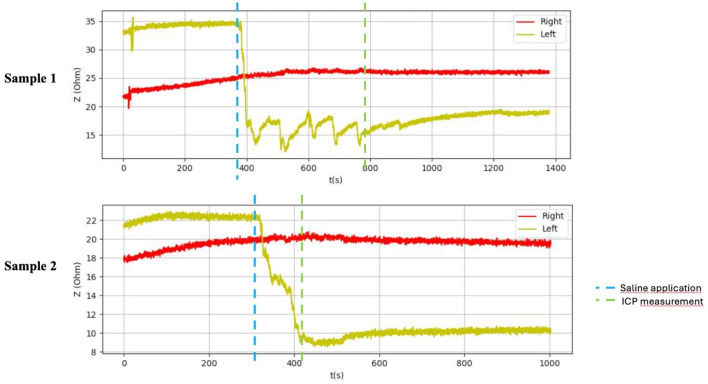
Graphical representation of the BACS measurement process (duck thighs).

**FIGURE 8:**
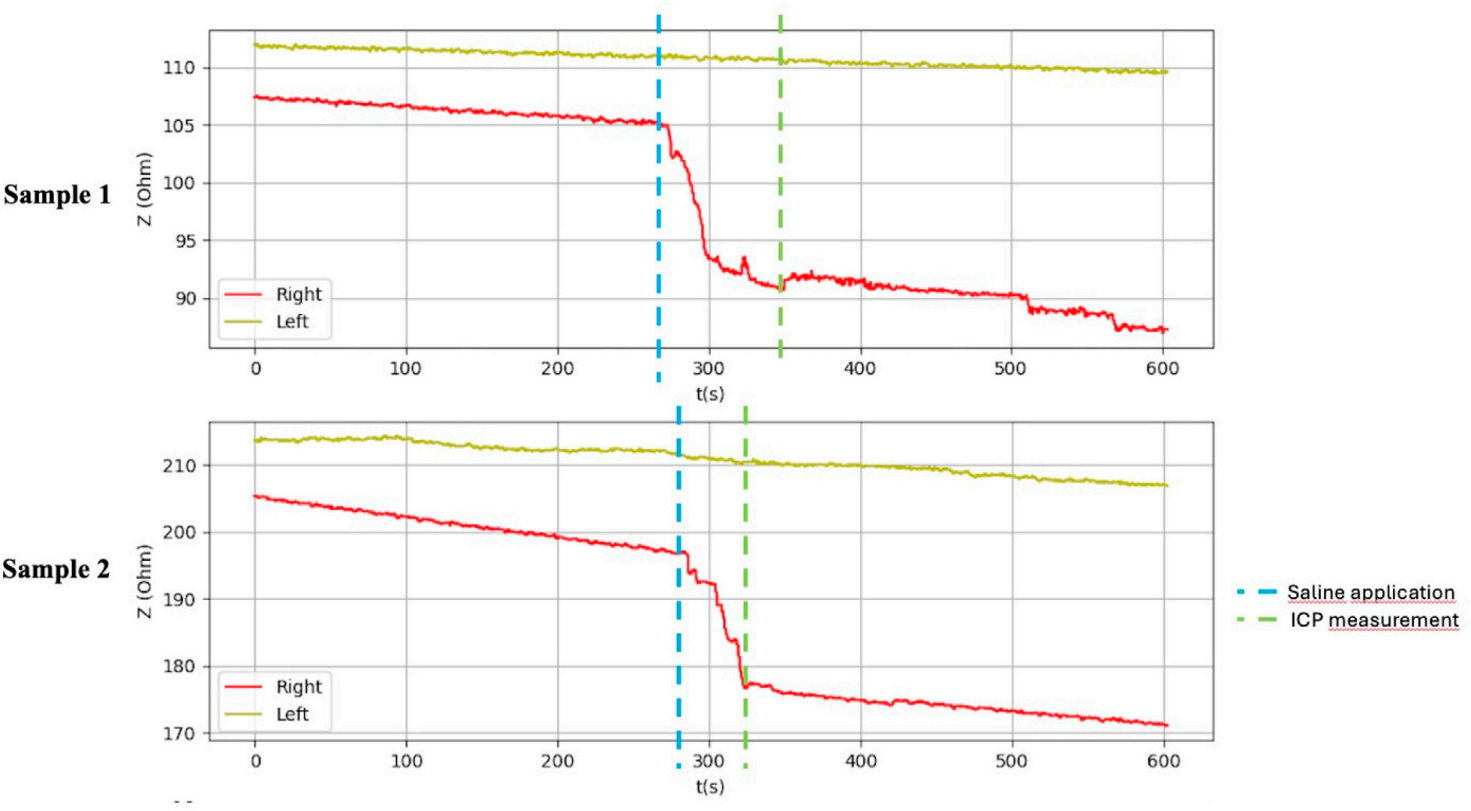
Graphical representation of the BACS measurement process (forelimb arms in the piglets).

**FIGURE 9:**
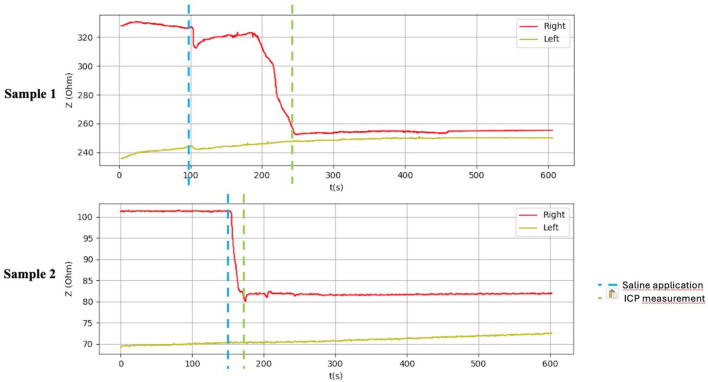
Graphical representation of the BACS measurement process (hindlimb thighs in the piglets).

After application of saline, the side with simulated compartment syndrome showed a decrease in the bioimpedance value for both samples from each inanimate biological material–chicken thighs −42, −35; duck breasts −43, −54; duck legs −20, −12; piglet forelimbs −16, −19; piglet hindlimbs −78, −27 Ω. (Table 1, Figures 6–9).

**TABLE 1:** Measurement results of the BACS prototype on inanimate biological material (L–left side, R–right side).

	Sample	Weight [kg]	Before/After application of saline			Amount of applied saline [ml]	Difference IMP [mmHg]	Difference impedance [Ω]
			IMP [mmHg]	Impedance [Ω]				
Chicken thighs	1	0,110	10/32	82/40 (L)	80/80 (R)	20	+22	−42(L)/0(R)
	2	0,120	11/45	69/34 (L)	65/68 (R)	40	+34	−35(L)/+3(R)
Duck breast	1	2,159	11/31	88/45 (R)	80/80 (L)	80	+20	−43(R)/0(L)
	2	2,175	13/34	94/40 (R)	95/88 (L)	100	+21	−54(R)/-7(L)
Duck legs	1	2,159	8/36	35/15 (L)	25/25 (R)	100	+28	−20(L)/0(R)
	2	2,175	10/34	22/10 (L)	18/20 (R)	120	+24	−12(L)/+2(R)
Piglets - forelimbs	1	20	15/42	108/92 (L)	113/110 (R)	20	+27	−16(L)/-3(R)
	2	21	17/45	101/82 (L)	69/72 (R)	25	+28	−19(L)/+3(R)
Piglets - hind limbs	1	20	12/35	330/252 (R)	238/250 (L)	60	+23	−78(R)/+12(L)
	2	21	13/38	205/178 (R)	215/210 (L)	50	+25	−27(R)/-5(L)

## Discussion

A non-invasive diagnosis of limb compartment syndrome based on bioimpedance measurements would be a breakthrough in the provision of healthcare not only for trauma patients. A non-invasive approach would allow continuous painless assessment of the developing compartment syndrome with minimal risk of secondary complications - bleeding, infection. The disadvantage is the limitation of use to patients who have sustained an injury to one of the paired limbs.

Bioimpedance measurement is used in clinical practice to record small changes in the impedance of organs that change their volume over time - heart, lungs, blood vessels (Khalil et al., 2014). A method referred to as impedance cardiography (ICG) is used to monitor the hemodynamic parameters of the heart and large vessels based on bioimpedance measurements as current passes through these structures. In this way, cardiac output and pulse volume can be non-invasively assessed in patients with heart failure (Krzenski et al., 2021). Furthermore, bioimpedance measurements are used in clinical practice to assess human body composition (percentage of body fat, total body water volume and the ratio between extracellular and intracellular fluid) (Campa et al., 2021).

Technically, bioimpedance is composed of resistance, which corresponds to the total amount of water in the human body, and reactance, which is a picture of the capacitance of cell membranes (Khalil et al., 2014). The reduction in cell membrane capacitance, which can be expected in the case of cell membrane destruction (cell death or its destruction directly by trauma), leads to a significant change in bioimpedance (Bera, 2014; Naranjo-Hernández et al., 2019). Mention of the use of bioimpedance measurements in limb compartment syndrome can be found in two studies (Tonkovic and Voloder, 1998; Tonkovic et al., 2000). The first study in 1998 was performed on 29 patients with a confirmed diagnosis of limb compartment syndrome. IMP and bioimpedance measurements were performed on both lower extremities. Values were compared between the injured and uninjured limb. On average, the injured limb had 18 mmHg higher IMP and 346 Ω lower impedance (Tonkovic and Voloder, 1998). Based on the results of our study, an absolute value IMP >30 mmHg was achieved in all 10 samples of inanimate biological material on the side with saline application (IMP variance 31–45 mmHg). Difference against the side without saline application was 20–34 mmHg (on average: 25,3 mmHg). In all cases of bioimpedance measurement, there was a decrease in bioimpedance after saline application in the compartment from 12 to 78 Ω (on average: 34,6 Ω). A greater average increase in IMP compared to the study from 1998 was likely due to nature of inanimate biological material, which is stiffer, and the application of even a smaller amount of saline leads to a larger increase in IMP. A smaller average decrease in bioimpedance compared to the referenced study was likely due to the absence of tissue destruction, which accompanies the actual development of compartment syndrome in patients. Tissue destruction leads to further release of intracellular fluid into the extracellular space, including ions that affect the bioimpedance value.

Table 1 shows the measurement results obtained during our study. The second sample of duck thighs and both samples of piglets’ front legs exhibit a smaller difference in impedance values. We believe that the IMP value and impedance value were evaluated within a shorter time interval after the application of the saline. In the graphical representation of the measurement progress on the piglets’ front legs (Figure 8), it is evident that impedance continued to decrease. We suspect that if we had evaluated impedance later, the difference would have been greater. At the moment when the amount of saline in the compartment stabilized, the bioimpedance value was already constant, however, it remained lower compared to the “healthy” side.

Various methods have been tested in the past for non-invasive diagnosis of compartment syndrome, such as tissue stiffness measurements, near-infrared spectroscopy (NIRS), elastography, and pulsed phase-locked loop (PPLL). However, their standard use in the diagnosis of compartment syndrome in clinical practice has not yet been published (Sellei et al., 2021). Measurement of tissue stiffness does not allow for continuous measurement but is praised for its ease of use. However, it suffers from low specificity and its readings can be significantly influenced by the amount of subcutaneous fat present in the patient. This makes it less reliable in providing accurate assessments of compartment syndrome. NIRS allows for continuous measurement, making it a valuable tool for ongoing monitoring. It is also easy to use, which adds to its practicality in clinical settings. Despite these advantages, NIRS has notable limitations. Its effective measurement depth is limited to a maximum of 3 cm, necessitating the use of a control compartment for accurate readings. Additionally, NIRS can be affected by factors such as skin pigment, hematomas, and defects in the integrity of the skin cover, potentially compromising its reliability. Elastography does not support continuous measurement but offers the advantage of not being affected by hematomas and subcutaneous fat. This can make it more reliable in certain scenarios compared to other methods that are influenced by these factors. However, its major drawback is that it has not yet been tested in patients with limb compartment syndrome, limiting the current understanding of its efficacy in this specific application. PPLL has potential benefits for continuous monitoring and ease application, similar to NIRS. Nevertheless, like elastography, it has not been tested in patients with limb compartment syndrome, raising questions about its applicability in clinical practice. Additionally, PPLL may be affected by low blood pressure, which can impact its accuracy and reliability in certain patients (Novak et al., 2022). The further valuable recent research advances in compartment syndrome can be found in (Peng et al., 2022; Yang et al., 2021; Karonen et al., 2021; Henn et al., 2021).

Advantage of the non-invasive method for diagnosing compartment syndrome by assessing changes in bioimpedance is its non-invasiveness and the minimization of secondary complications such as infection and bleeding, and potential subjective perception of pain. Clearly, the bioimpedance value responds to the presence of saline in the compartment. It can be inferred that the change in bioimpedance could be monitored and observed over a longer timeline in the increasing swelling of the limb after trauma, when there is an accumulation of extracellular fluid in the compartment space. A further decrease in bioimpedance might also be expected based on the reduction in capacitance that is caused by the destruction of cell membranes after trauma.

In the experimental study conducted, it was shown that the small distance between the compartments being compared leads to the compartments influencing each other. However, no influence was observed when comparing bioimpedance on paired limbs. It was also found that the smaller Kendall CA610 ECG electrodes were more suitable for bioimpedance measurements, as they have better adhesion to inanimate biological material and can be used on smaller areas to be measured (e.g., in paediatric patients).

A current limitation of the non-invasive bioimpedance measurement method is the necessity for comparative measurement. This method cannot be applied to patients who have had an amputation on one limb and have compartment syndrome on the other paired limb. However, developments suggest that tetrapolar bioimpedance measurement may eventually be feasible without the need for comparative measurement. Another limitation of this measurement technique is the requirement for an area where measuring electrodes must be applied. Although the use of Kendall CA610 electrodes allows for the technique to be applied to smaller areas, it is important to note that the skin should be intact. This means further clinical research is needed to verify whether this technique can be used, for example, in patients after fasciotomy, or with skin injuries. A relative limitation for the application of this method is the immobilization of the limb, such as a plaster splint, in which windows can be created to attach the measuring electrodes.

## Conclusion

The measurement results of our study clearly demonstrated a change in bioimpedance when the extracellular fluid was increased. We can conclude that the idea of measuring bioimpedance and its changes in compartment syndrome proved to be correct. The application of the prototype BACS device in the non-invasive diagnosis of the limb compartment syndrome in clinical practice requires further clinical testing.

## Data Availability

The datasets presented in this article are not readily available because the dataset will be released upon a request on a corresponding author. Requests to access the datasets should be directed to jan.kubicek@vsb.cz.

## References

[B1] Bera T. K. (2014). Bioelectrical impedance methods for noninvasive health monitoring: a review. J. Med. Eng. 2014, 1–28. 10.1155/2014/381251 PMC4782691 27006932

[B2] Campa F. Toselli S. Mazzilli M. Gobbo L. A. Coratella G. (2021). Assessment of body composition in athletes: a narrative review of available methods with special reference to quantitative and qualitative bioimpedance analysis. Nutrients 13 (5), 1620. 10.3390/nu13051620 34065984 PMC8150618

[B3] Henn J. Lingohr P. Branchi V. Semaan A. Von Websky M. W. Glowka T. R. (2021). Open abdomen treatment in acute pancreatitis. Front. Surg. 7, 588228. 10.3389/fsurg.2020.588228 33521045 PMC7841327

[B4] Jagminas L. (2022). Compartment pressure measurement. Medscape.

[B5] Karonen E. Wrede A. Acosta S. (2021). Risk factors for fasciotomy after revascularization for acute lower limb ischaemia. Front. Surg. 8, 662744. 10.3389/fsurg.2021.662744 33855045 PMC8039517

[B6] Kassanos P. (2021). Bioimpedance sensors: a tutorial. IEEE Sens. J. 1-1, 22190–22219. 10.1109/JSEN.2021.3110283

[B7] Khalil S. F. Mohktar M. S. Ibrahim F. (2014). The theory and fundamentals of bioimpedance analysis in clinical status monitoring and diagnosis of diseases. Sensors 14 (6), 10895–10928. 10.3390/s140610895 24949644 PMC4118362

[B8] Krzenski P. Sobotnicki A. Gacek A. Siebert J. Walczak A. Murawski P. (2021). Noninvasive bioimpedance methods from the viewpoint of remote monitoring in heart failure. JMIR Mhealth Uhealth 9 (5), 25937. 10.2196/25937 PMC8135018 33949964

[B9] Mubarak S. J. Owen C. A. Hargens A. R. Garetto L. P. Akeson W. H. (1978). Acute compartment syndromes: diagnosis and treatment with the aid of the wick catheter. Surg. 60, 1091–1095. 10.2106/00004623-197860080-00012 721856

[B10] Naranjo-Hernández D. Reina-Tosina J. Min M. (2019). Fundamentals, recent advances, and future challenges in bioimpedance devices for healthcare applications. J. Sens. 2019, 1–42. 10.1155/2019/9210258

[B11] Novak M. Penhaker M. Raska P. Pleva L. Schmidt M. (2022). Extremity compartment syndrome: a review with a focus on non-invasive methods of diagnosis. Front. Bioeng. Biotechnol. 10, 801586–802022. 10.3389/fbioe.2022.801586 35923576 PMC9340208

[B12] Peng B. Wan T. Tan W. Guo W. He M. (2022). Novel retrograde tibial intramedullary nailing for distal tibial fractures. Front. Surg. 9, 899483. 10.3389/fsurg.2022.899483 35620192 PMC9127322

[B13] Piuzzi E. Pisa S. Pittella E. Podestá L. Sangiovanni S. (2018). Low-cost and portable impedance plethysmography system for the simultaneous detection of respiratory and heart activities. IEEE Sens. J. 19 (7), 2735–2746. 10.1109/JSEN.2018.2887303

[B14] Sellei M. Kobbe R. Hildebrand F. (2021). Non-invasive diagnostics in acute compartment syndrome. London, United Kingdom: IntechOpen. 10.5772/intechopen.97874

[B15] Tonkovic S. Tonkovic I. Kovacic D. (2000). Bioelectric impedance analysis of lower leg ischaemic muscles. 22nd Annu. Int. Conf. IEEE Eng. Med. Biol. Soc. (cat. No.00CH37143) 1, 757–760. 10.1109/IEMBS.2000.900859

[B16] Tonkovic S. Voloder D. Compartmental syndrome diagnostics using custom designed bioimpedance analyzer. , 1998, vol. 2, 1480–1484. 10.1109/MELCON.1998.699486

[B17] Yang E. Chan S.-Y. C. Al-Omari Y. Ward L. Yap T. E. Jhass A. (2021). A functional radiological and soft tissue classification to predict outcomes in orbital fracture Surgery in a multidisciplinary “real-world” setting. Front. Surg. 8, 693607. 10.3389/fsurg.2021.693607 34386516 PMC8353087

